# A Complex Relationship Between Suicide, Dementia, and Amyloid: A Narrative Review

**DOI:** 10.3389/fnins.2018.00371

**Published:** 2018-06-01

**Authors:** Ismael Conejero, Sophie Navucet, Jacques Keller, Emilie Olié, Philippe Courtet, Audrey Gabelle

**Affiliations:** ^1^Department of Psychiatry, Caremeau Hospital, University Hospital of Nîmes, Nîmes, France; ^2^Inserm U1061, Neuropsychiatry: Epidemiological and Clinical Research, La Colombière Hospital, University of Montpellier, Montpellier, France; ^3^Centre de Biochimie Structurale, University of Montpellier, Montpellier, France; ^4^Department of Montpellier, Memory Resources Research Center, Gui De Chauliac Hospital, University of Montpellier, Montpellier, France; ^5^Department of Psychiatric Emergency and Post-Acute Care, Lapeyronie Hospital, University of Montpellier, Montpellier, France

**Keywords:** amyloid, Alzheimer's dementia, decision-making, suicide, depression

## Abstract

**Background:** Suicide rates are high among older adults and many conditions have been related to suicide in this population: chronic illnesses, physical disabilities, cancer, social isolation, mental disorders and neurocognitive disorders.

**Objectives:** Among neurocognitive disorders, analysis of the relationships between dementia and suicidal behaviors led to conflicting results and some questions are still without answer. Particularly, it is not known whether (i) Alzheimer's disease (AD) increases the risk of suicidal ideation and suicide attempts (SA) or the frequency of death by suicide; (ii) the presence of suicidal ideation or SA in people older than 65 years of age is an early dementia sign; and (iii) amyloid load in frontal areas facilitates SA by modifying the decision-making pathway.

**Methods:** Therefore, in this narrative review, we searched the PubMed database using the medical subject heading (MeSH) terms (“Suicide” AND “Depression”) OR (“Amyloid” OR “Dementia”) to identify recent (from 2000 to 2017) original studies on the links between suicidal behavior, dementia and brain amyloid load. We also explored the clinical and pathophysiological role of depression in these relationships.

**Results and Discussion:** The findings from these studies suggest that late stage dementia could protect against suicidal ideation and SA. Conversely, the risk of complete suicide is increased during the early phase of cognitive decline.

**Conclusions:** Serious cognitive impairment and decline of executive functions could protect against negative thoughts related to cognitive disability awareness and against suicide planning.Several factors, including brain amyloid load, could be involved in the increased suicide rate early after the diagnosis of dementia.

## Introduction

Suicide is a major public health issue and the 13 cause of death worldwide. Suicidality can be represented as a continuum from suicidal ideation to suicidal act, which includes suicide attempts (SA) and death by suicide. Although SA frequency is higher in young adults (Conejero et al., 2016) and then progressively decreases (Hawton and Harriss, 2008), suicide rates increase with age, reaching the highest level in older adults in almost all countries (World Health Organization, 2014). Indeed, suicide rate among white men older than 85 years of age was 48.7/100,000 in the United States in 2004 (more than four times the national age-adjusted rate of 11.1/100,000), and 140/100,000 among men aged or older than 75 in rural China in 1999 (Conwell and Thompson, 2008). Moreover, among older people, suicide rate increases with age (Shah et al., 2016). The ratio between deliberate self-harm and completed suicide varies from 200 for teenagers to 10 for people over 60 (Hawton and Harriss, 2008). In older adults, many conditions have been related to suicide: chronic illnesses, physical disabilities, cancer, social isolation, mental and neurocognitive disorders (Duberstein et al., 2004a,b; Voshaar et al., 2015). Among neurocognitive disorders, the study of the relationships between dementia and suicidal behaviors gave conflicting results. Dementia represents the major cause of autonomy loss in older adults. As a chronic disease, it may induce depressive symptoms and suicidal ideation. Alzheimer's disease (AD), the most frequent cause of dementia, is characterized clinically by cognitive dysfunctions, most commonly involving episodic memory and behavioral disorders. AD pathogenesis is thought to be driven by pathological aggregation of beta amyloid (Aβ) and tau proteins in the brain (Reitz et al., 2011; Uzun et al., 2011). Aβ deposition seems to be the first brain lesion, particularly in frontal areas. Several techniques are used to assess brain amyloid burden, including brain positron emission tomography (PET) and biomarker quantification in cerebrospinal fluid (CSF) (Uzun et al., 2011). *In vivo* biomarker quantification could allow the diagnosis of AD at an early stage when only cognitive complaints are present, or even earlier in people at risk due to family history or harboring the apolipoprotein Eε4 allele (APOE4). AD diagnosis could represent a critical moment that increases the risk of suicidal ideation and act. In addition, some early behavioral disorders in AD, such as depression and altered decision-making related to frontal brain lesions, may contribute to increase the suicide risk. Specifically, it is not clear whether (i) AD increases the risk of suicidal ideation and SA, or the frequency of death by suicide; (ii) the presence of suicidal ideation or SA in older (≥65-year-old) people could be an early sign of AD; and (iii) the amyloid load in frontal areas facilitates SA by modifying the decision-making pathway. In this review, we provide updated findings on the links between suicidal behavior, dementia and brain amyloid burden, in order to address these questions. We also discuss the clinical and pathophysiological role of depression in the relationship between dementia and suicidal behaviors.

## Materials and methods

**Figure 1:**
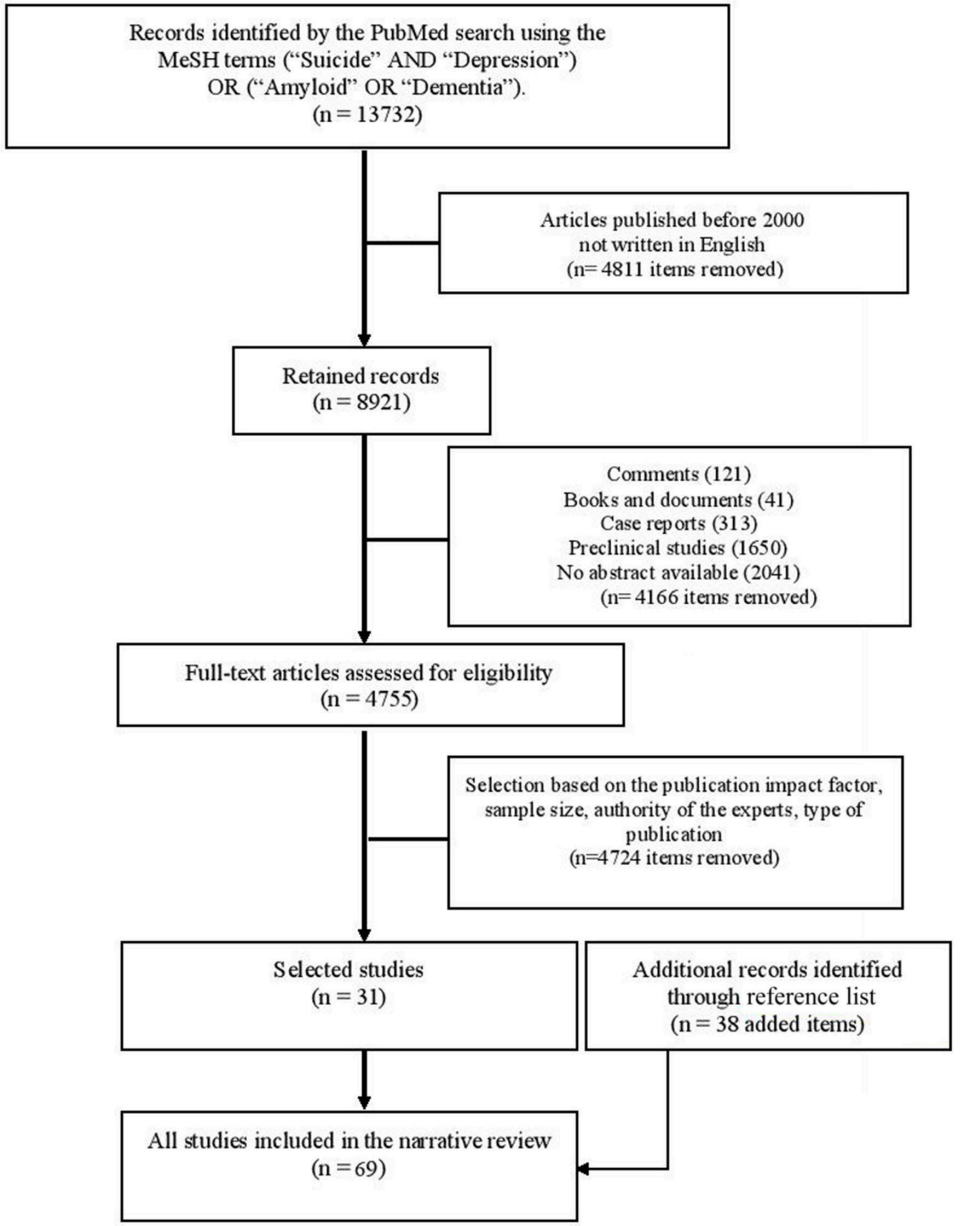
Chart presenting the selection process of the studies.

We conducted a narrative review of original studies selected from PubMed using the medical subject heading (MeSH) terms (“Suicide” AND “Depression”) OR (“Amyloid” OR “Dementia”). Among the 13,732 articles retrieved, we retained 8,921 written in English, after 2000. We excluded comments, books, documents, case reports, preclinical studies and articles with no abstract available. Then, we kept 4,755 full-text articles. Finally, we selected 31 articles written in English that were published from 2000 to 2017 and corresponded to the most representative studies (e.g., in terms of impact factor, sample size, authority of the experts, type of publication, such as meta-analyses/reviews). The list of references was also reviewed to identify other studies of interest (*n* = 38). The study flow chart is presented in Figure 1.

## Results and discussion

### Suicide and dementia

#### Association between completed suicide and dementia

There are conflicting results in the literature concerning the link between completed suicide and dementia. These discrepancies could be explained by the heterogeneity of the dementia groups, the lack of standardization of the tools used for the diagnosis of dementia and suicidal behaviors, and also the absence of stratification relative to the disease stage. Although accurate histopathological assessment was performed in few works, retrospective studies do not allow the precise categorization of the dementia type based only on the standardized clinical and para-clinical examination. Furthermore, most of the studies on suicide and dementia were performed in relatively small clinical samples.

##### Presence of association

Patients with dementia have a (3- to 10-fold) higher risk to die by suicide, even when taking into account potential confounding factors, such as mood disorders (Erlangsen et al., 2008). Among the different dementia types, patients with Huntington's disease are particularly at risk (Harris and Barraclough, 1997; Haw et al., 2009), with a rate of completed suicide of about 13% (Cummings, 1995). In women, the suicide risk associated with vascular dementia is significantly lower than that associated with AD (Erlangsen et al., 2008). AD pathology (based on examination of hippocampal sections) is more frequent in people older than 60 years of age who committed suicide (n = 28) than in age- and sex-matched controls (*n* = 56) (Rubio et al., 2001). Specifically, the modified Braak score (reflecting the number of neurofibrillary tangles) was higher in the suicide victims than in controls (Rubio et al., 2001), whereas amyloid load was comparable in both groups.

Moreover, during the early stages of AD, the risk of completed suicide is highest and then decreases (Erlangsen et al., 2008; Cipriani et al., 2013). In the first 6 months after the diagnosis of dementia, the increased risk for completed suicide could be explained by the: (1) awareness of cognitive decline (Serafini et al., 2016) and feeling of burdensomeness toward significant others; (2) stress induced by the anticipation of autonomy loss and the feeling of impairment in daily life functioning (Cipriani et al., 2013); (3) increased prevalence of comorbid depressive and adjustment disorders (Seyfried et al., 2011; Cipriani et al., 2013; Draper, 2015) (4) effect of potential comorbidities, such as bipolar disorders, substance use, and anxiety disorders (Seyfried et al., 2011); (5) still good cognitive functions at the early stage of disease that allow patients to plan suicide and complete the suicidal act; and (6) deficits of executive functions, decision-making and inhibition process (Richard-Devantoy et al., 2012, 2016). Other risk factors for completed suicide have been identified at the early AD stage, such as late onset of cognitive decline, male gender and high educational level (Cipriani et al., 2013). However, the role of each of these factors has not been validated in patients in whom AD diagnosis was confirmed by neuropathological data or *in vivo* biomarker quantification. In addition, the clinical characteristics of early stage AD differed among studies, and no data was available on prodromal AD symptoms.

##### Absence of association

Some studies did not find any additional risk of completed suicide in people with dementia. For instance, a comparison of 85 cases of suicide and 153 living controls older than 65 years found that the odds ratio for the association with dementia is less than one (Wærn et al., 2002). No completed suicide was observed among the 104 participants who died (76 were women) during the four-year follow-up of 277 patients with dementia (Harris and Barraclough, 1997). The analysis of hippocampal sections of 143 community-dwelling suicide victims aged over 65 years and 59 controls did not highlight any difference in plaque score and neurofibrillary tangle staging (Peisah et al., 2007). The American Psychiatric Association guidelines on the care of patients with dementia have reported no added risk of suicide in this population “*Elderly persons in general and elderly men in particular are at increased risk for suicide, although the diagnosis of dementia is not known to confer added risk”, p. 18* (Rabins and McIntyre, 2010). Severe cognitive impairment in late stage dementia could protect against completed suicide by reducing the capacity to accomplish a suicidal plan (Cipriani et al., 2013).

#### Association between dementia and suicide attempt or suicidal ideation

In individuals with dementia, SA rate is lower than 1% (Schneider et al., 2001) and even completely absent (retrospective analysis of 148 patients with AD, 24 with vascular dementia and 49 with dementia types) (Draper et al., 1998). It seems that SA occurs in patients with dementia and psychiatric comorbidities (Draper, 2015). In patients older than 65 years of age hospitalized in a psychiatric unit following SA, SA was positively associated with comorbid psychiatric disorders and history of SA. Moreover, the risk for suicidal behaviors decreases when cognitive impairment increases, based on to the Mini Mental State Examination (MMSE) score (Osvath et al., 2005).

Suicidal ideation in patients with dementia is rare. Based on the suicide item (item 3) of the Hamilton Rating Scale for Depression, 10% (*n* = 9) of 91 patients with probable AD reported hopelessness, but not suicidal ideation (Harwood and Sultzer, 2002). Lifetime suicidal ideas are not more frequent in patients with dementia than in age-matched non-demented participants, even after controlling for lifetime major depression. In addition, suicidal ideation and feelings of worthlessness are correlated with the severity of cognitive decline, measured by the MMSE (Heun et al., 2003). In another study, almost 4% of patients with AD reported suicidal ideation (“wish to die” by 3.2% and suicidal ideation by 0.9%, according to the Hamilton Rating Scale for Depression) (Draper et al., 1998).

The apparent contradiction between studies showing that dementia could predispose to complete suicide, especially at an early stage of the disease, and other works reporting that dementia could be associated with a lower risk for SA or suicidal ideation could partly explained by the impact of the diagnosis announcement and the long-term management of the disease. This is an important point because AD diagnosis could be realized at an early stage and patient education and awareness on this issue is constantly increasing. Therefore, it is crucial to improve our knowledge about the potential triggering effects of the announcement of a diagnosis of dementia (Mattsson et al., 2010; Mitchell et al., 2013). Although this could have a positive impact, such as deciding to spend more time with loved ones, forming partnerships with other people with dementia, receiving therapeutic support, improving the quality of patient care (De Lepeleire et al., 2004), the effect on suicidal ideation/act needs to be further assessed. To our knowledge, very few studies have directly analyzed the association between dementia diagnosis disclosure and suicidal behavior. Turnbull et al. (2003) evaluated the attitudes toward AD diagnosis in 200 outpatients older than 65 years of age and found that 92% wanted to know about the diagnosis and 1.7% wanted to be told about the diagnosis of AD in order to commit suicide (Turnbull et al., 2003). However, the impact of AD diagnosis disclosure on suicide risk should be more specifically assessed.

Studies show a suicidal ideation prevalence between 2.2 and 16.7% in Chinese aging populations (Simon et al., 2013), and more than 5% in other representative samples of older adults (Barnow and Linden, 2000; Scocco and De Leo, 2002). In our clinical experience, the rate of suicidal ideation and SA in individuals with cognitive complaints after the first visit to a memory center is lower than 2%. In a feasibility study, we assessed suicidal ideation/act using the C-SSRS questionnaire (Posner et al., 2011) in patients with cognitive complaints at the Memory Resources Research Center of Montpellier from January 31, 2016 to July 1, 2017. Among the 1691 participants, only 32 [1.9%, median age 61 (45–88) years] had at least one positive answer to the following two questions: “Have you wished you were dead or wished you could go to sleep and never wake up?” and “Have you really thought about committing suicide?” We classified participants as having moderate suicidal ideation (*n* = 16, 0.95%) when they gave only one positive answer, and as having severe suicidal ideation (*n* = 16, 0.95%) when they gave two positive answers. Among these 32 participants, 21.8% had past history of SA, 31.2% had a neurodegenerative disorder (AD, frontotemporal dementia, Lewy body disease) and 31.2% had a subjective memory complaint. Among the participants with negative answers to both questions, only 0.4% reported past history of SA (*unpublished data*).

#### Are suicidal behavior and amyloid associated via depression?

No study has analyzed the relationship between the underlying pathological process, such as the amyloid load, and suicidal behaviors. Depression could induce suicidal ideation and/or SA, but also brain amyloid deposition or changes in the CSF level of amyloid peptides. For instance, in a group of ≥60-year-old people without dementia, amyloid burden, measured by PET with 18-F-florbetapir, in the parietal and precuneus cortices was higher among people with lifetime diagnosis of major depressive disorder than in controls without depression (Wu et al., 2014). These amyloid deposits are related to treatment-resistant depression (Li et al., 2017), a well-known risk factor of suicidal behavior (Greden, 2001; Olin et al., 2012). Depression is often found in patients with early stage dementia and could be a consequence of neurobiological changes in specific brain regions (Andersen et al., 2005). In patients with mild cognitive impairment (MCI), amyloid load has been linked to late-onset depression (Tateno et al., 2015), and to lifetime history of depression (Chung et al., 2015). Depression level has been associated with higher FDDNP binding to amyloid plaques and neurofibrillary tangles (quantified by PET) in the lateral temporal regions in participants with MCI, and with FDDNP binding in the medial temporal cortex in controls without MCI (Lavretsky et al., 2009). Moreover, FDDNP binding in the posterior cingulate and lateral temporal regions is higher in depressed than healthy controls aged between 60 and 82 years (Kumar et al., 2011). Apathy severity, evaluated with the Apathy Evaluation Scale, has been associated with amyloid load (by FDDNP-PET) in the anterior cingulate cortex in 16 patients with late-life depression (Eyre et al., 2017). Conversely, using the Pittsburgh Compound-B (PiB) tracer, findings are controversial (Butters et al., 2008; Madsen et al., 2012; Yasuno et al., 2016). Most studies detected a significant association between Aβ burden and depressive symptoms or major depressive episodes; however, some negative findings were also reported, possibly due to the small sample size and lack of statistical power. Concerning CSF biomarkers, changes in Aβ42 level or Aβ40/42 ratio have been related to late-life depression (Nascimento et al., 2015). During a longitudinal follow-up, participants with depression displayed a slightly, but significant lower CSF Aβ42 level than non-depressed individuals (Pomara et al., 2016). CSF Aβ42 may be a state-dependent marker. Indeed, Aβ42 levels are lower in more severe depression, and the improvement of depressive symptoms is associated with CSF Aβ42 increase (Pomara et al., 2016).

The higher amyloid burden during depression in older people is associated with increased risk to develop AD in the future; however, it is difficult to distinguish between depression as a prodromal manifestation of dementia, or as independent condition. Additional studies with longitudinal design, amyloid PET imaging or CSF amyloid measurements, and depression assessment are needed to determine the potential causal relationship. Indeed, depression could be a risk factor (Barnes et al., 2012), or a prodromal manifestation of dementia, particularly amyloid-associated depression (Sun et al., 2008).

Amyloid deposition in the central nervous system has also been linked to serotoninergic dysregulation, dysfunctional stress response, and inflammation of brain regions involved in suicidal vulnerability. Serotoninergic pathways have been involved in Aβ-associated depressive episodes and are impaired by Aβ accumulation in the central nervous system (Gonzalo-Ruiz et al., 2003). Moreover, the serotoninergic system is altered in AD (Trillo et al., 2013; Ramirez et al., 2014; Verdurand and Zimmer, 2017), and amyloid deposition in the brain impairs the serotoninergic activity in animal models (Colaianna et al., 2010; Ledo et al., 2013). Aβ peptides display neurotoxic activity (Piccinni et al., 2013), and they could also alter the brain inflammatory response, which in turn modifies the expression of indoleamine2,3-dioxygenase and impairs serotoninergic transmission (Ledo et al., 2013; Mahgoub and Alexopoulos, 2016). Hence, in early stage AD, suicidal behavior could be related to alterations of serotoninergic transmission (Madsen et al., 2011), which have been linked to impulsive and aggressive behavior (Lai et al., 2003; Vermeiren et al., 2014). In addition, Aβ could impair the stress response (Catania et al., 2009; Morgese et al., 2017) and dysregulate the hypothalamic-pituitary-adrenal axis that has been involved in suicidal vulnerability (Jokinen and Nordström, 2008; Jokinen et al., 2010; Morgese et al., 2014).

Finally, decision-making impairment (i.e., the choice of options with high immediate reward, but disadvantageous in the long-term) has been observed in early stage AD (Gleichgerrcht et al., 2010), especially in patients with amygdala neurodegeneration and altered connectivity to the ventromedial prefrontal cortex. Moreover, impaired decision-making has been linked to CSF amyloid level in patients with Lewy body disease, and to medial orbitofrontal cortex atrophy (Spotorno et al., 2017). However, to our knowledge, the relationship between decision-making impairment and amyloid load in AD has not been evaluated. Impaired decision-making has been also involved in the pathophysiology of suicidal behavior in older adults. Using the Cambridge Gamble Task (Clark et al., 2011), a study showed that among ≥65-year-old patients, the quality of decision-making was reduced in depressed suicide attempters compared with depressed non-attempters and healthy controls. Altered decision-making was associated with perceived poor social problem-solving. In this study, impaired decision-making in older suicide attempters might not be related to the level of impulsivity, but rather to neglected knowledge of probability. Similarly, low performances at the Iowa Gambling Task have been observed in violent suicide attempters over 65 years of age (Wyart et al., 2016). These results raise the possibility that amyloid deposition could trigger suicidal behavior by altering decision-making in patients with early stage AD.

#### Strengths and limitations

The present narrative review of the literature provides an updated picture of the most recent findings concerning the link between suicidal ideation, suicidal behaviors and dementia. However, some limitations should be underlined. First, most of the studies have been conducted on small population samples with clinical heterogeneity according to the age and the disease stage. Second, a very few studies explored the link between suicide and the different types of dementia. Hence, we rather focused on the link between suicide and AD. Finally, because our aim was to provide an updated overview of the literature, we did not integrated results published before 2000 in our review.

## Conclusion

The presented results suggest that at late disease stages, dementia could protect against suicidal ideation and SA. Conversely, the risk of complete suicide is increased during the early phase of cognitive decline. Serious cognitive impairment and decline of executive functions might protect against negative thoughts related to awareness of cognitive disability and against suicide planning. Several factors could contribute to increasing the suicide rate following the diagnosis of dementia: (1) the awareness of cognitive decline and the feeling of burdensomeness toward significant others, (2) the anticipation of autonomy loss, (3) the increased prevalence of comorbid mood and adjustment disorders, (4) the still good cognitive functions at the early stage of disease that allow patients to plan and complete a suicidal act; (5) the deficit of executive functions, decision-making and inhibition process.

However, such retrospective analyses do not allow highlighting any clear causal relationship between dementia and suicidal behavior. Indeed, suicidal acts in older adults may trigger or precipitate cognitive decline by increasing the stress response and activation of the hypothalamic-pituitary-adrenal axis. However, some findings suggest that SA or completed suicide in patients with early stage AD could be a consequence and a complication of the neurocognitive impairment.

**Figure 2:**
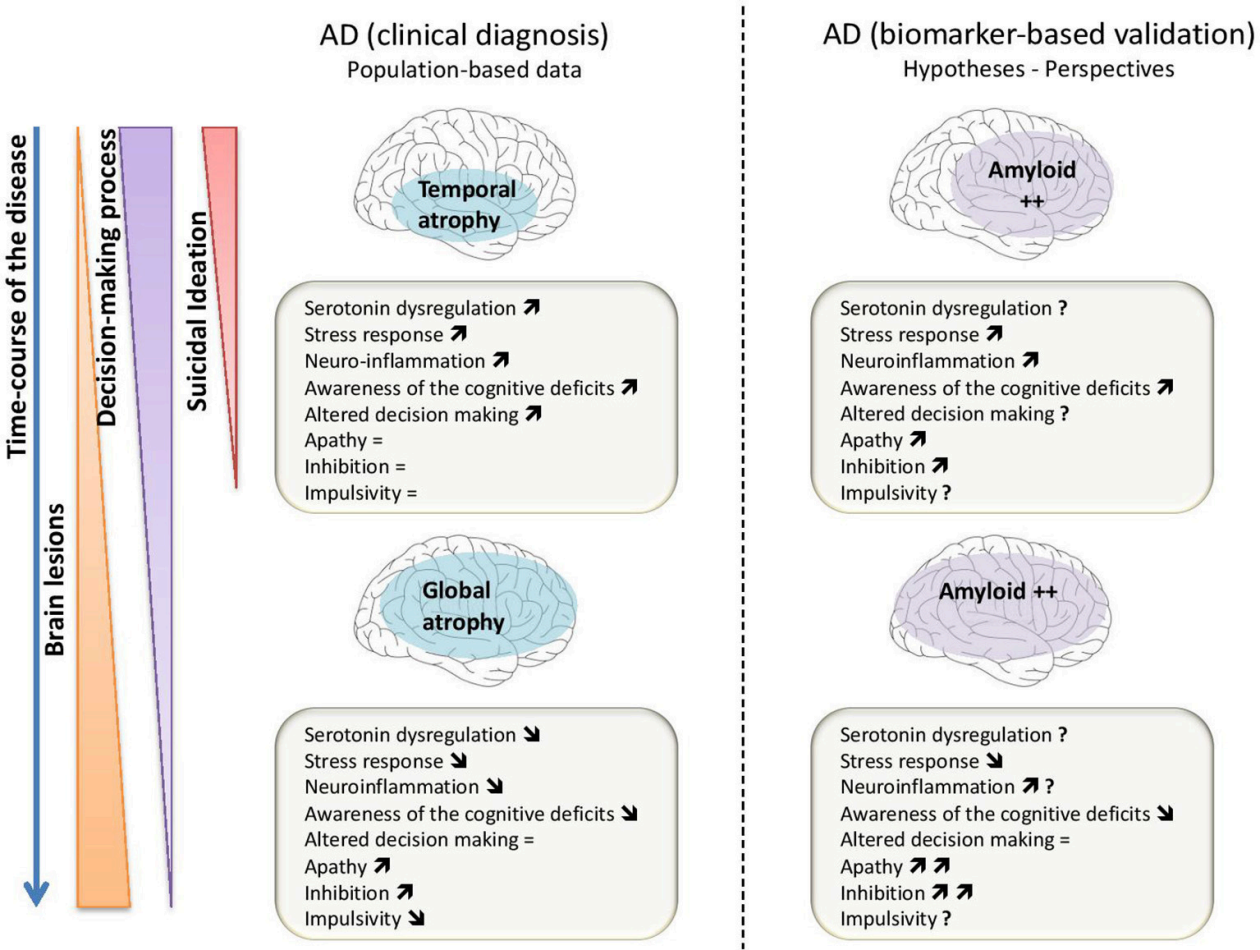
Scheme of the different mechanisms that could be involved to explain the relationship between suicidal ideation, decision-making process in Alzheimer's disease (AD) based on clinical diagnosis (part left of the figure) and in AD based on biomarkers especially the amyloid load (part right of the figure). The “↑” is for an increase of the mechanism or pattern; the “↓” is for a decrease and “ = ” is related to a stability or an absence of modification of the mechanism or pattern; the “?” is for an unknown data or mechanism.

Amyloid burden is a potential risk factor for suicide through its association with depressive symptoms that are frequently observed in the early stage of dementia, and through its effects on various neurobiological pathways (i.e., serotoninergic dysregulation, dysfunctional stress response and brain inflammation) (Figure 2). Some evidences suggest that Aβ load directly alters some of the most important neurobiological pathways underlying suicidal behavior. The suicidal behavior observed in older people early after the diagnosis of dementia should encourage research to assess implicit behavioral data in this population in order to improve suicidal behavior prevention.

## Author contributions

IC, SN, JK, EO, AG analyzed the data of the literature and drafted the manuscript. PC, AG conceived and designed the review.

### Conflict of interest statement

The authors declare that the research was conducted in the absence of any commercial or financial relationships that could be construed as a potential conflict of interest.
